# Lifestyle Factors, Sociodemographic Characteristics and Incident Hypertension: A Prospective Analysis of the Korean National Health Insurance Service Sample Cohort

**DOI:** 10.3390/jpm14090959

**Published:** 2024-09-09

**Authors:** Jung-Eun Lee, Anthony Kityo, Sang-Ah Lee

**Affiliations:** 1 Interdisciplinary Graduate Program in Medical Bigdata Convergence, Kangwon National University School of Medicine, Chuncheon 24341, Republic of Korea; je5645@kangwon.ac.kr; 2 Department of Preventive Medicine, Kangwon National University School of Medicine, Chuncheon 24341, Republic of Korea; akkityo@kangwon.ac.kr

**Keywords:** hypertension, smoking, drinking, physical activity, lifestyle, sociodemographic

## Abstract

Background: Hypertension is a significant chronic disease globally, and lifestyle modifications are crucial for the prevention of this disease. We conducted a longitudinal analysis of the associations between lifestyle factors and the incidence of hypertension, stratified by sociodemographic characteristics. Methods: We analyzed 113,022 adults (65,315 men), aged 20 years or older from the Korean National Health Insurance Service-National Sample Cohort 2.0 who participated in health screening between 2002 and 2003. Lifestyle factors (smoking, drinking, physical activity) were assessed at baseline using self reports, and incident hypertension was defined based on physician diagnoses. Cox proportional hazards regression models were used to assess associations. Results: During an 11.6-year follow-up, 26,812 new cases of hypertension were identified. The risk of hypertension was high among men and women who smoked over 20 cigarettes daily (men: hazard ratio [HR]: 1.15; 95% confidence interval [CI], 1.08–1.21; women: HR: 1.62; 95% CI 1.17–2.25) and those who drank over 1.5 bottles of alcohol daily (men, HR: 1.18; 95% CI, 1.12–1.24; women, HR: 1.23; 95% CI 1.02–1.47). These associations tended to be high in high-income men (HR: 1.09; 95% CI, 1.04–1.14), low-income women (HR: 1.19; 95% CI, 1.05–1.35) and non-obese women (HR: 1.13; 95% CI, 1.01–1.27) who currently smoked. Physical activity was inversely associated with incident hypertension in men (HR: 0.96; 95% CI, 0.93–0.99). Conclusions: Unhealthy lifestyle factors, such as heavy smoking and drinking, was associated with an increased risk of hypertension, with variations by income, BMI, and sex. These findings underscore the importance of tailored, population-specific prevention strategies to address hypertension disparities.

## 1. Introduction

Hypertension is a major risk factor for cardiovascular disease (CVD) and mortality [1]. Since 1990, the prevalence of hypertension is gradually increasing [2] and is projected to increase with population growth and aging. The prevalence of hypertension is estimated at 45.4% in the United States [3], and approximately 12 million patients were identified with hypertension in 2019 in South Korea [4].

Hypertension is influenced by both modifiable lifestyle factors and sociodemographic characteristics. Modifiable lifestyle factors include smoking, alcohol consumption, low physical activity, obesity, and sodium intake [5,6,7,8,9]. Previous studies have reported various associations between lifestyle factors and hypertension. For example, smoking is implicated in CVD risk factors, including hypertension [10]. Previous studies reported a high prevalence of hypertension among former smokers [11], and an increased risk of hypertension has been reported among light and heavy drinkers in men and women, respectively [12]. Regular physical activity may have a beneficial effect on hypertension, but results have been inconsistent [13]. Moreover, there are some reports on sociodemographic determinants of hypertension, such as residential area and income level [14,15,16].

Previous studies were conducted as cross-sectional designs in Western countries, making it difficult to confirm the association between lifestyle factors and hypertension considering socio-demographic factors. Therefore, this study aimed to conduct a longitudinal analysis of the associations between lifestyle factors—specifically, smoking, alcohol consumption, and physical activity—and the incidence of hypertension, stratified by sociodemographic characteristics, within a representative cohort of Korean adults.

## 2. Materials and Methods

### 2.1. Database and Study Population

A population-based cohort study was conducted using the Korean National Health Insurance Service-National Sample Cohort (NHIS-NSC 2.0) database of over 1 million participants. The NHIS was introduced in 1977 to provide healthcare coverage to all Korean citizens. Since 1989, all citizens have been registered with the NHIS [17]. The NHIS collects information on medical use and prescriptions through hospital records. In June 2017, the NHIS-NSC 2.0 database was constructed [18]. This database contains information on insurance premiums, medical diagnoses, medication history, drug prescriptions, death records, demographic information, biochemical measurements, and physical examination from January 2002 to December 2015 for a random sample of over 1 million people, representing 2% of the nation’s population eligible for health insurance as of 2006. A detailed description of this cohort can be found elsewhere in the literature [19].

**Figure 1:**
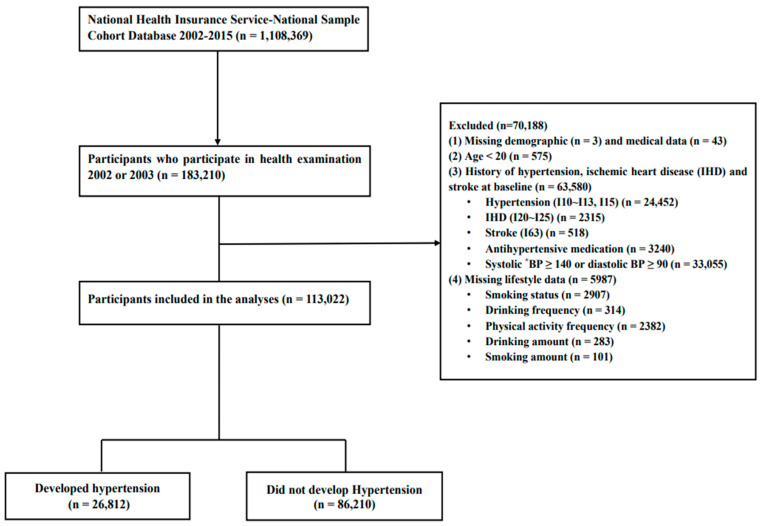
Flowchart showing selection of study participants. * BP, blood pressure.

Out of 1,108,369 participants in the NHIS-NSC 2.0, 183,210 who underwent at least one general health examination between 2002 and 2003 were selected. Participants with missing demographic or medical information (*n* = 46); under the age of 20 years (*n* = 575); and with pre-existing hypertension (*n* = 24,452), ischemic heart disease (*n* = 2315), and stroke (*n* = 518) were excluded. Furthermore, participants with a history of antihypertensive medication use (*n* = 3240), those with systolic blood pressure ≥ 140 mmHg or diastolic blood pressure ≥ 90 mmHg (*n* = 33,055) [20], and those with missing data on lifestyle variables of interest (*n* = 5987) at baseline were excluded, leaving 113,022 participants (65,315 men) for analysis (Figure 1).

This study was reviewed and approved by the Institutional Review Board (IRB) of Kangwon National University (IRB No. KWNUIRB-2021-11-004-002. Approval date: 26 July 2022) and the National Health Insurance Corporation (NHIS) (No. NHIS-2022-2-123). This study was conducted according to the guidelines of the Declrkdaration of Helsinki.

### 2.2. Outcome

Hypertension was defined based on the disease codes of the 10th revision of the International Classification of Diseases (ICD-10-I10) [21] and having a billing history of one or more claims of antihypertensive medication at least once a year. The medications used in the definition of hypertension were diuretics; alpha, beta, and calcium channel blockers; angiotensin-converting enzyme inhibitors; and angiotensin II receptor blockers [22].

### 2.3. Exposure

Smoking, drinking, and physical activity were assessed using a self-reported questionnaire. Smoking was categorized based on responses to the question: “How often do you smoke?” as never smokers, past smokers (smoked in the past but quit), or current smokers (still smoke). Current smokers were further categorized based on responses to “How many cigarettes do you smoke per day?” into 3 groups: light smokers (smoking < 10 cigarettes), moderate smokers (smoking 10–19 cigarettes), heavy smokers (smoking ≥ 20 cigarettes). Drinking frequency was categorized as non-drinker, less than once a week, 1–2 times a week, 3–4 times a week, and 5–7 times a week, based on the question, “What is your drinking habit?”. Drinking amount was categorized as non-drinker, 0.5 bottles per day, 1 bottle per day, and 1.5 bottles or more per day based on the question, “If you drink, how much do you drink per occasion?”. Drinking was defined based on soju [18], the most commonly consumed alcoholic beverage in Korea [23]. Soju is a distilled beverage. Physical activity was defined as none, 1–2 times per week, and 3–4 times per week or more based on the question, “How many times per week do you engage in physical activity that makes you sweat?”.

### 2.4. Sociodemographic and Health-Related Factors

Sociodemographic characteristics included sex, age (<40, 40–49, 50–59, >60 years), residential area (urban: Seoul, Busan, Incheon, Daegu, Gwangju, Daejeon, Ulsan, Sejong Special Self-Governing City; rural: other cities and provinces), income level (high: 6th–10th quintile, low: 0–5th quintile), insurance type (employee-insured, self-insured). Physical factors and medical conditions were collected from the health examination database and included weight, height, and blood pressure. Height and weight were measured and accurately recorded by trained staff. Body mass index (BMI) was calculated as the weight divided by the square of height (kg/m^2^) and classified as BMI < 25 kg/m^2^, BMI ≥ 25 kg/m^2^. Other medical conditions were defined as follows: presence of chronic diseases (diabetes: yes or no; kidney disease: yes or no; cancer: yes or no) and family history of hypertension (yes or no).

### 2.5. Statistical Analysis

Person-years were calculated starting from the date of health examination (2002–2003) to the date of first diagnosis of hypertension, use of hypertension medication, loss of insurance eligibility (emigration or death), or censoring date (31 December 2015), whichever came first. To select any potential confounders, we built a Cox model with age, residential area, income level, insurance type, BMI, and family history of hypertension as independent variables and hypertension as a dependent variable, separately for men and women. Those selected variables were used as covariates in Cox models evaluating the association of lifestyle factors with hypertension, stratifying by sex. Model 1 was adjusted for age, and Model 2 was further adjusted for residential area, income level, insurance type, BMI, family history of hypertension, other medical conditions (diabetes, kidney disease, cancer), and drinking amount and mutually adjusted for lifestyle factors. Finally, models stratified by sociodemographic factors and BMI were built based on Model 2.

All statistical analyses were performed using the SAS software version 9.4 (SAS Institute, Cary, NC, USA), and the significance level was set at *p* < 0.05.

### 2.6. Ethics Statement

This study was reviewed and approved by the Institutional Review Board (IRB) of Kangwon National University (IRB No. KWNUIRB-2021-11-004-002) and the National Health Insurance Corporation (NHIS) (No. NHIS-2022-2-123). This study was conducted according to the guidelines of the Declaration of Helsinki.

## 3. Results

During a mean follow-up of 11.6 years, 26,812 cases of hypertension occurred. Associations of general characteristics of participants with hypertension risk are presented in Table 1. Age, rural residence, self-insurance, obesity, and pre-existing diabetes were positively associated with hypertension in both men and women, but high income in men and low income in women were inversely associated with hypertension.

Associations between lifestyle factors and hypertension risk in men and women are presented in Table 2. Compared with never-smokers, past and current smokers were 1.10 times and 1.07 times, respectively, more likely to develop hypertension in men. However, smoking > 20 cigarettes per day was associated with an increased risk of hypertension in men (HR = 1.15) and women (HR = 1.62). Drinking frequency was associated with a graded increase in the risk of hypertension, with the highest risk observed among men (HR = 1.30) and women (HR = 1.43) who drank 5–7 times/week compared to non-drinkers. In terms of drinking amount, the risk of hypertension was high in men who drank ≥ 1 bottle/day (HR = 1.18) and women who drank ≥ 1 bottle/day (HR = 1.23). Men who were physically active had a 4% reduced risk of developing hypertension compared to men who were not physically active.

The positive association of smoking with hypertension risk was only observed in men in their 40s (Table 3). The increased risk of hypertension with drinking was primarily seen in men younger than 60 years, particularly in those who drank ≥ 3 times/week. Physical activity was inversely associated with hypertension among men in their 40s and those ≥ 60 years old and in women aged 50 years.

The association of lifestyle factors and incident hypertension according to sociodemographic characteristics is presented in Table 4. Among men, past smoking was positively associated with incident hypertension among high-income earners, those with obesity, rural residents, and those on employee insurance policies. Current smoking was associated with a higher risk of hypertension regardless of BMI or insurance type, but associations were more pronounced among high-income earners (HR = 1.09) and urban residents (HR = 1.13). For alcohol consumption, the risk was higher among men regardless of income level and obesity status and was particularly high among urban residents and the employee-insured. However, among physically active men, a decreased risk of hypertension was observed in high-income earners, rural residents, and the self-employed. In women, current smoking was associated with a higher risk of hypertension in low-income earners and those of normal weight.

## 4. Discussion

This study examined the sex-specific associations between lifestyle factors and hypertension among 113,022 adults and found that heavy smoking (≥20 cigarettes/day), frequent drinking (5–7 times/week), and drinking ≥ 1.5 bottles/day were positively associated with an increased risk of developing hypertension in both men and women. However, physical activity was associated with a reduced risk of hypertension only in men.

Previous studies reported an association between smoking and the risk of hypertension, but the results have been inconsistent [24]. Although some studies have shown that the blood pressure of current smokers is lower or not different from that of never-smokers [11], most studies have shown that smoking causes a temporary increase in blood pressure [10] and that heavy smoking is a major determinant of hypertension, regardless of sex [25]. A study conducted in China found that smoking ≥ 20 cigarettes/day was associated with a higher risk of developing hypertension compared to never-smokers [26]. In addition, a positive association between smoking and hypertension was observed in a study of Japanese male workers [27]. This trend was similar for women as well [28]. Nicotine in cigarette smoke stimulates the sympathetic nervous system, which can cause a temporary increase in blood pressure, leading to hypertension [29].

A positive association between drinking and hypertension has been reported in previous epidemiologic studies [30,31]. A meta-analysis found that light-to-moderate drinking of 1–2 cups per day was associated with an increased risk of hypertension in men; however, this association was only true for women when they drank 2 or more cups per day [12]. In a Korean study, high alcohol intake (men ≥ 30 g/day, women ≥ 15 g/day) was associated with an increased risk of developing hypertension in both men and women [32]. Occasional or frequent binge drinkers had 11% and 24% higher risks of hypertension, respectively, compared with non-drinkers [33]. A possible mechanism by which excessive alcohol affects blood pressure is impairment of endothelial cell function [34]. As a result, most health guidelines recommend stopping or minimizing alcohol consumption [22].

In this study, the risk of hypertension was significantly reduced in men who were physically active. Physical activity reduces the risk of developing hypertension [35], and higher levels of moderate or vigorous physical activity have a greater protective effect against hypertension [36]. In a previous study that identified a sex-specific association between physical activity and hypertension, moderate or low levels of occupational physical activity in women increased their risk of developing hypertension by approximately 36–38% [37]. In addition, a meta-analysis of randomized controlled clinical trials showed that moderate-intensity leisure-time physical activity significantly reduced systolic and diastolic blood pressure and heart rate [13]. The decrease in blood pressure with physical activity can be explained by a reduction in sympathetic nerve activity and a decreased release of norepinephrine that mediates vasoconstriction [38]. Therefore, this study indicates that regular physical activity may help in preventing hypertension.

Additionally, unhealthy lifestyle factors such as smoking, alcohol consumption, and low physical activity are closely associated with chronic inflammation, which can contribute to the development of hypertension [21,39,40]. Previous studies have shown that smoking promotes arterial stiffness, leading to increased blood pressure [41], while alcohol consumption can damage the vascular endothelium, reducing vasodilators like nitric oxide and subsequently raising blood pressure [42]. Physical activity is associated with skeletal muscle function, which plays a critical role in generating and releasing proteins such as cytokines during muscle contraction, and this process helps to reduce systemic inflammation [43]. Thus, low physical activity is directly linked to increased levels of pro-inflammatory cytokines, exacerbating inflammation-related pathophysiological conditions such as hypertension, dyslipidemia, and endothelial dysfunction [44].

Similar to previous studies, a positive association was observed between lifestyle and hypertension risk according to age [45]. The risk of hypertension particularly increased in men in their 40s who had smoked and in men under 60 who drank alcohol three or more times per week. These findings may be explained by the increased risk of developing hypertension due to work stress [46]. High job stress has been reported to worsen health and contribute to cardiovascular disease, including hypertension [47,48]. Middle-aged or elderly men are primarily workers and experience high levels of work stress and pressure. This leads to smoking and heavy drinking, which can contribute to the development of hypertension [49]. However, this study did not examine questions related to work stress, so further research is needed in this area. Moreover, engaging in physical activity 1–2 times per week reduced the risk of hypertension in middle-aged and older adults, which is consistent with previous studies reporting the benefits of physical activity in preventing hypertension in this population [50].

When analyzing the association between lifestyle and hypertension risk by income level, a significant increase in the risk of developing hypertension was observed in high-income men and low-income women with unhealthy lifestyles. A previous study reported an association between income and hypertension, and a study conducted in Vietnam found a higher risk of hypertension in wealthier men despite lower levels of education or occupational status and in poorer women with lower levels of occupational status [51]. In addition, women of lower socioeconomic status were found to be at a higher risk of developing hypertension [52]. These differences can be interpreted as differences in lifestyle based on income level. Low-income people are more likely to consume more salt, have unhealthy lifestyles such as smoking and drinking, and have difficulty accessing medical services, which may be associated with hypertension [53,54]. In contrast, high-income earners are reported to generally adopt healthier lifestyles, such as healthier diets and higher levels of physical activity, compared with low-income earners. However, some studies have shown that high-income earners consume more salt [55] and are at higher risk of developing hypertension due to psychological pressure at work or sedentary jobs [56]. These findings highlight sex-specific differences in lifestyle and hypertension risk based on income, suggesting the need for tailored health interventions for the prevention of hypertension.

On the other hand, in an analysis considering BMI, the risk of hypertension was higher in female smokers who were not obese. These results could be attributed to the fact that smoking is generally associated with lower body weight. Smoking suppresses appetite and increases energy expenditure, leading to weight loss [57]. Moreover, smokers, in particular, are more concerned about their weight [25]. Younger female smokers tend to start smoking to control their weight, and smokers who perceive themselves as overweight or have concerns about their weight are more likely to choose smoking as a way to control their weight [58]. In addition, adults trying to lose weight are more likely to smoke [59] and have an unhealthy diet [60]. Therefore, these results suggest that the interaction between smoking and body weight may influence the development of hypertension.

In addition, hypertension risk was found to be higher among male smokers living in urban areas than among never-smokers, which is consistent with previous studies that have reported a higher prevalence of hypertension in urban rather than rural areas in several countries [61,62,63]. This trend can be explained by urbanization, which has been reported to be strongly associated with the prevalence of hypertension [64]. Moreover, a study conducted in Malawi, Sub-Saharan Africa, found that the prevalence of overweight, obesity, and hypertension is higher among people who migrated from rural to urban areas [65]. This can be explained by lifestyle changes, such as decreased physical activity and alterations in diet [66]. In addition, people living in urban areas tend to have more sedentary jobs and consume more high-fat and animal-based foods, which can increase the prevalence of overweight and obesity, which in turn increases the risk of developing hypertension [14,67]. Hence, these findings suggest the need for comprehensive policies to improve diet and lifestyle in urban areas.

Finally, male smokers and drinkers on employee insurance had a higher risk of developing hypertension. Hypertension in workers can be triggered by long hours of work, physical inactivity, and work-related stress [68], which is further supported by extremely long working hours in South Korea per week [69]. Long hours of working have a negative impact on health [70]. A study of young Korean workers found that working long hours increased the risk of stress and depression [71]. This high stress can lead to unhealthy behaviors such as excessive smoking and drinking, physical inactivity, and insomnia, which are, in turn, associated with the development of hypertension [72,73,74,75]. Therefore, education and health campaigns are needed to educate workers about the dangers of smoking and alcohol consumption, emphasizing the need to switch to a healthy lifestyle.

Several limitations of this study should be considered. First, these data may overestimate certain chronic conditions due to disease codes created solely for health insurance claims. To minimize this, we defined hypertension based on both physician diagnosis and prescribed antihypertensive medication. Second, the NHIS-NSC 2.0 Database does not assess educational level, stress, and diet; hence, we could not account for other important risk factors for hypertension. Additionally, important anthropometric factors related to hypertension, such as chest wall conformation, could not be considered. A recent study has shown that patients with concave-shaped chest wall conformation or various degrees of anterior chest wall conformation generally have a lower prevalence of hypertension and a lower risk of coronary artery disease than those with normal chest wall conformation. They generally have a narrow anterior and posterior chest diameter and are often observed in women. In addition, the burden of cardiovascular disease, including hypertension, is low and has a good prognosis during the mid-to-long-term follow-up period [76,77]. Further research is needed for this. Third, there might have been coding errors or redundant entries from the data source. Finally, this study was conducted on a Korean population, so further research involving different ethnic groups or countries is necessary to generalize the findings. Despite these limitations, this study has several strengths. First, this study used the NHIS-NSC 2.0 database, which is representative of the general Korean population. Second, a longitudinal analysis of the incidence of hypertension in South Koreans allowed us to infer causal conclusions. Third, by stratifying the analysis by demographic factors that may influence hypertension, we could identify lifestyle risk factors that influence hypertension based on demographic characteristics. Lastly, lifestyle factors were categorized by frequency and amount to more accurately assess their impact on the risk of developing hypertension.

## 5. Conclusions

High daily smoking, high weekly alcohol consumption, and drinking 1.5 bottles or more per day were found to be strong risk factors for hypertension in both men and women. In contrast, physical activity was shown to reduce hypertension risk in men. These associations varied according to income, BMI, residential area, and insurance type, demonstrating that maintaining an unhealthy lifestyle may particularly increase the risk of hypertension in high-income men and low-income or non-obese women. These findings indicate that hypertension risk is influenced by lifestyle habits across all income groups, not just the traditionally studied low-income populations. This highlights the need for multi-faceted customized interventions, policies, and public health strategies to prevent hypertension, depending on the characteristics of the population.

## Figures and Tables

**Table 1:** General characteristics of the participants with hypertension according to sex.

Characteristic	Men (*n* = 14,611)				Women (*n* = 12,201)			
	Person-Years	Events	Model 1	Model 2	Person-Years	Events	Model 1	Model 2
Age (years)								
<40	442,244.6	3981	1.00	1.00	257,182.1	1820	1.00	1.00
40–49	207,703.5	4634	2.51 (2.40–2.62)	2.32 (2.22–2.42)	172,699.9	4245	3.49 (3.31–3.69)	3.16 (2.98–3.35)
50–59	79,977.4	3263	4.68 (4.46–4.90)	4.25 (4.05–4.46)	72,829.8	3100	6.10 (5.75–6.46)	5.16 (4.85–5.49)
≥60	39,454.8	2733	8.25 (7.86–8.67)	7.55 (7.16–7.96)	40,967.4	3036	10.80 (10.19–11.45)	8.99 (8.43–9.58)
Residential area								
Urban	348,761.7	6387	1.00	1.00	261,215.1	5268	1.00	1.00
Rural	420,618.6	8224	1.07 (1.03–1.10)	1.06 (1.03–1.10)	282,464.1	6933	1.22 (1.18–1.26)	1.08 (1.04–1.12)
Income level								
Low	299,367.9	5009	1.00	1.00	304,714.6	6008	1.00	1.00
High	470,012.4	9602	1.22 (1.18–1.27)	1.05 (1.01–1.09)	238,964.6	6193	1.32 (1.27–1.36)	0.93 (0.90–0.96)
Insurance								
Employee-insured	663,375.1	10,864	1.00	1.00	411,841.5	7138	1.00	1.00
Self-insured	106,005.2	3747	2.19 (2.11–2.28)	1.15 (1.10–1.20)	131,837.7	5063	2.22 (2.14–2.30)	1.12 (1.08–1.17)
BMI (kg/m^2^)								
<25	546,727.2	9061	1.00	1.00	452,737.7	8387	1.00	1.00
≥25	222,653.1	5550	1.51 (1.46–1.56)	1.56 (1.51–1.61)	90,941.5	3814	2.27 (2.19–2.36)	1.51 (1.45–1.57)
Diabetes								
No	749,536.5	13,377	1.00	1.00	530,353.7	11,350	1.00	1.00
Yes	19,843.8	1234	3.62 (3.41–3.84)	2.01 (1.89–2.13)	13,325.5	851	3.01 (2.81–3.23)	1.55 (1.45–1.67)
Kidney disease								
Yes	276.2	13	1.00	1.00	244.4	10	1.00	1.00
No	769,104.1	14,598	0.39 (0.23–0.67)	0.65 (0.38–1.12)	543,434.8	12,191	0.55 (0.29–1.02)	0.88 (0.47–1.64)
Cancer								
No	761,733.0	14,292	1.00	1.00	535,277.3	11,880	1.00	1.00
Yes	7647.3	319	2.27 (2.03–2.54)	1.20 (1.07–1.34)	8401.9	321	1.72 (1.54–1.93)	1.16 (1.04–1.30)
Family history of hypertension								
No	721,439.3	13,541	1.00	1.00	502,998.0	11,350	1.00	1.00
Yes	47,941.0	1070	1.19 (1.12–1.27)	1.33 (1.24–1.41)	40,681.2	851	0.93 (0.87–0.99)	1.11 (1.03–1.19)

BMI, body mass index. Diabetes (ICD-10-E10~E14). Model 1: non-adjusted. Model 2: adjusted for age, residential area, income level, insurance, BMI, family history of hypertension, other disease (diabetes, kidney, cancer).

**Table 2:** Association between lifestyle and hypertension.

Lifestyle Factors	Men (*n* = 14,611)				Women (*n* = 12,201)			
	Person-Years	Events	Model 1	Model 2	Person-Years	Events	Model 1	Model 2
Smoking Status								
Never-smoker	256,985.7	5236	1.00	1.00	520,541.1	11,637	1.00	1.00
Ex-smoker	107,751.7	2308	1.12 (1.07–1.18)	1.10 (1.05–1.16)	8199.0	155	1.06 (0.90–1.24)	1.04 (0.88–1.22)
Current-smoker	404,642.9	7067	1.08 (1.04–1.12)	1.07 (1.03–1.12)	14,939.1	409	1.11 (1.00–1.22)	1.08 (0.97–1.19)
Smoking amount								
Never/Past-smoker	364,737.4	7544	1.00	1.00	528,740.1	11,792	1.00	1.00
Light smoker (<0/day)	87,289.3	1597	0.97 (0.92–1.02)	0.97 (0.92–1.03)	9982.3	259	1.03 (0.91–1.16)	1.00 (0.89–1.14)
Moderate smoker (10–19/day)	236,621.2	3676	1.04 (1.00–1.08)	1.03 (0.99–1.08)	4170.2	114	1.18 (0.98–1.42)	1.14 (0.95–1.38)
Heavy smoker (≥20/day)	80,732.4	1594	1.16 (1.10–1.23)	1.15 (1.08–1.21)	786.6	36	1.73 (1.25–2.40)	1.62 (1.17–2.25)
Drinking frequency								
Non-drinker	224,844.7	4859	1.00	1.00	376,870.4	9587	1.00	1.00
<1/week	213,269.1	3122	0.96 (0.92–1.01)	1.07 (1.00–1.14)	109,805.6	1600	0.98 (0.93–1.03)	1.15 (0.95–1.39)
1–2/week	235,815.3	4084	1.09 (1.04–1.13)	1.17 (1.11–1.24)	47,430.1	782	1.11 (1.03–1.19)	1.28 (1.06–1.55)
3–4/week	71,266.5	1642	1.18 (1.12–1.25)	1.24 (1.16–1.32)	7048.3	141	1.12 (0.95–1.33)	1.22 (0.97–1.54)
5–7/week	24,184.7	904	1.23 (1.15–1.32)	1.30 (1.20–1.41)	2524.8	91	1.30 (1.06–1.60)	1.43 (1.10–1.87)
Drinking amount								
Non-drinker	224,844.7	4859	1.00	1.00	376,870.4	9587	1.00	1.00
≤0.5 bottle/day	143,040.1	2788	0.96 (0.92–1.00)	0.97 (0.92–1.01)	113,822.8	1921	1.00 (0.95–1.05)	1.01 (0.96–1.06)
1 bottle/day	269,152.5	4704	1.11 (1.06–1.15)	1.11 (1.06–1.16)	44,513.7	572	1.10 (1.01–1.20)	1.08 (0.99–1.18)
≥1.5 bottle/day	13,234.3	2260	1.20 (1.14–1.27)	1.18 (1.12–1.24)	8472.3	121	1.27 (1.06–1.52)	1.23 (1.02–1.47)
Physical activity								
No	361,441.8	7400	1.00	1.00	371,539.4	8351	1.00	1.00
Yes	407,938.5	7211	0.96 (0.93–1.00)	0.96 (0.93–0.99)	172,139.8	3850	0.98 (0.95–1.02)	0.97 (0.94–1.02)

Model 1: adjusted for age. Model 2: adjusted for residential area, income level, insurance, BMI, family history of hypertension, other disease (diabetes, kidney, cancer), and drinking amount.

**Table 3:** Association of lifestyle factors by age group with hypertension.

Age Group	Men (*n* = 14,611)				Women (*n* = 12,201)			
	Person-Years	Events	Model 1	Model 2	Person-Years	Events	Model 1	Model 2
<40								
Smoking status								
Never-smoker	131,897.0	1119	1.00	1.00	244,251.1	1716	1.00	1.00
Past-smoker	54,563.9	535	1.16 (1.04–1.28)	1.09 (0.98–1.21)	5295.5	39	1.05 (0.77–1.44)	1.04 (0.76–1.44)
Current-smoker	255,783.7	2327	1.07 (1.00–1.15)	1.05 (0.97–1.13)	7635.5	65	1.22 (0.95–1.56)	1.16 (0.89–1.50)
Drinking frequency								
Never-drinker	112,144.2	941	1.00	1.00	146,702.7	1087	1.00	1.00
<1/week	141,611.3	1188	1.00 (0.92–1.09)	1.05 (0.94–1.17)	75,050.8	478	0.86 (0.77–0.96)	0.98 (0.73–1.31)
1–2/week	147,221.6	1409	1.14 (1.05–1.24)	1.17 (1.05–1.30)	30,981.0	219	0.96 (0.83–1.10)	1.07 (0.79–1.44)
≥3/week	41,267.5	443	1.28 (1.15–1.44)	1.28 (1.13–1.45)	4447.6	36	1.10 (0.79–1.53)	1.08 (0.72–1.62)
Physical activity								
No	204,197.4	1853	1.00	1.00	185,498.4	1323	1.00	1.00
1–2/week	166,206.9	1484	0.98 (0.92–1.05)	0.97 (0.91–1.04)	48,621.2	321	0.93 (0.82–1.05)	0.92 (0.81–1.03)
≥3–4/week	71,840.3	644	0.99 (0.90–1.08)	0.96 (0.87–1.05)	23,062.5	176	1.07 (0.91–1.25)	1.05 (0.89–1.23)
40–49								
Smoking status								
Never-smoker	72,596.8	1512	1.00	1.00	166,808.7	4070	1.00	1.00
Past-smoker	35,105.8	826	1.13 (1.04–1.23)	1.10 (1.01–1.20)	1770.7	49	1.14 (0.86–1.51)	1.04 (0.78–1.39)
Current-smoker	10,000.9	2296	1.10 (1.04–1.18)	1.09 (1.02–1.17)	4120.5	126	1.26 (1.06–1.50)	1.09 (0.91–1.31)
Drinking frequency								
Never-drinker	62,373.7	1384	1.00	1.00	131,894.6	3223	1.00	1.00
<1/week	50,632.1	983	0.87 (0.80–0.95)	0.93 (0.84–1.04)	25,094.6	593	0.97 (0.89–1.06)	1.25 (0.91–1.71)
1–2/week	62,960.0	1398	1.00 (0.93–1.08)	1.02 (0.93–1.13)	12,193.0	325	1.09 (0.97–1.22)	1.35 (0.98–1.85)
≥3/week	31,737.7	869	1.24 (1.14–1.35)	1.21 (1.09–1.34)	3517.7	104	1.21 (1.00–1.47)	1.32 (0.95–1.84)
Physical activity								
No	94,063.3	2203	1.00	1.00	110,025.0	2744	1.00	1.00
1–2/week	73,890.8	1566	0.90 (0.85–0.96)	0.89 (0.84–0.95)	33,629.5	824	0.98 (0.91–1.06)	1.01 (0.94–1.10)
≥3–4/week	39,749.4	865	0.93 (0.86–1.00)	0.89 (0.82–0.97)	29,045.4	677	0.93 (0.86–1.02)	0.95 (0.88–1.04)
50–59								
Smoking status								
Never-smoker	33,546.2	1321	1.00	1.00	70,608.4	2988	1.00	1.00
Past-smoker	12,801.9	556	1.11 (1.00–1.22)	1.09 (0.98–1.20)	682.7	32	1.10 (0.78–1.56)	1.02 (0.72–1.45)
Current-smoker	33,629.3	1386	1.05 (0.97–1.13)	1.02 (0.94–1.10)	1538.7	80	1.23 (0.99–1.54)	1.18 (0.94–1.48)
Drinking frequency								
Never-drinker	30,964.0	1206	1.00	1.00	61,408.1	2563	1.00	1.00
<1/week	15,772.3	624	1.02 (0.92–1.12)	1.20 (1.04–1.38)	7082.2	338	1.15 (1.02–1.28)	1.11 (0.69–1.79)
1–2/week	19,210.1	826	1.11 (1.01–1.21)	1.26 (1.11–1.44)	3254.0	155	1.14 (0.97–1.34)	1.12 (0.69–1.82)
≥3/week	14,031.0	607	1.11 (1.01–1.22)	1.21 (1.06–1.38)	1085.5	44	0.97 (0.72–1.30)	0.91 (0.54–1.55)
Physical activity								
No	39,606.4	1664	1.00	1.00	46,180.6	2047	1.00	1.00
1–2/week	23,738.4	933	0.93 (0.86–1.01)	0.95 (0.88–1.03)	13,461.3	519	0.87 (0.79–0.96)	0.89 (0.81–0.98)
≥3–4/week	16,632.6	666	0.95 (0.87–1.04)	0.94 (0.86–1.03)	13,187.9	534	0.91 (0.83–1.00)	0.92 (0.84–1.01)
≥60								
Smoking status								
Never-smoker	18,945.7	1284	1.00	1.00	38,872.9	2863	1.00	1.00
Past-smoker	5280.1	391	1.09 (0.97–1.22)	1.08 (0.96–1.21)	450.1	35	1.06 (0.76–1.47)	1.04 (0.74–1.46)
Current-smoker	15,229.0	1058	1.02 (0.94–1.11)	1.06 (0.97–1.15)	1644.4	138	1.14 (0.96–1.35)	1.13 (0.95–1.34)
Drinking frequency								
Never-drinker	19,362.8	1328	1.00	1.00	36,865.0	2714	1.00	1.00
<1/week	5253.4	327	0.90 (0.80–1.02)	0.84 (0.69–1.03)	2578.0	191	1.01 (0.87–1.17)	1.04 (0.46–2.35)
1–2/week	6423.6	451	1.02 (0.92–1.14)	0.96 (0.79–1.15)	1002.1	83	1.13 (0.90–1.40)	1.15 (0.50–2.62)
≥3/week	8415.0	627	1.09 (0.99–1.20)	1.02 (0.86–1.21)	522.3	48	1.25 (0.94–1.66)	1.30 (0.56–3.01)
Physical activity								
No	23,574.7	1680	1.00	1.00	29,835.4	2237	1.00	1.00
1–2/week	7218.7	446	0.87 (0.78–0.96)	0.87 (0.78–0.97)	4817.9	339	0.94 (0.84–1.05)	0.94 (0.84–1.05)
≥3–4/week	8661.4	607	0.98 (0.90–1.08)	0.96 (0.87–1.06)	6314.1	460	0.97 (0.88–1.07)	0.94 (0.85–1.04)

Model 1: non-adjusted. Model 2: adjusted for residential area, income level, insurance, BMI, family history of hypertension, other disease (diabetes, kidney, cancer), and drinking amount.

**Table 4:** Hazard ratios of hypertension according to lifestyle factors stratified by sociodemographic factors and BMI.

Lifestyle	Men (*n* = 14,611)						Women (*n* = 12,201)					
	Income Level						Income Level					
	Low			High			Low			High		
	Person- Years	Events	Adjusted HR	Person-Years	Events	Adjusted HR	Person-Years	Events	Adjusted HR	Person- Years	Events	Adjusted HR
Smoking status												
Past-smoker	32,023.6	617	1.01 (0.92–1.11)	75,728.1	1691	1.14 (1.07–1.21)	5442.8	88	1.06 (0.86–1.31)	2756.2	67	1.02 (0.80–1.30)
Current-smoker	175,591.6	2677	1.04 (0.98–1.11)	229,051.3	4390	1.09 (1.04–1.14)	10,388.1	268	1.19 (1.05–1.35)	4551.0	141	0.93 (0.79–1.10)
Drinking status (drinker)	208,439.2	3194	1.07 (1.01–1.14)	336,096.4	6558	1.07 (1.02–1.12)	111,315.1	1535	1.04 (0.97–1.10)	55,493.7	1079	1.02 (0.95–1.09)
Physical activity (Yes)	148,404.0	2221	0.97 (0.92–1.03)	259,534.5	4990	0.96 (0.92–0.99)	85,879.6	1644	0.98 (0.92–1.03)	86,260.2	2206	0.99 (0.94–1.04)
	BMI						BMI					
	<25			≥25			<25			≥25		
Smoking status												
Past-smoker	72,293.7	1345	1.09 (1.02–1.16)	35,458.0	963	1.09 (1.01–1.18)	6969.9	114	1.05 (0.87–1.26)	1229.1	41	1.04 (0.76–1.42)
Current-smoker	292,151.2	4419	1.06 (1.01–1.11)	112,491.7	2648	1.14 (1.07–1.21)	12,291.8	305	1.13 (1.01–1.27)	2647.3	104	0.96 (0.79–1.17)
Drinking status (drinker)	384,083.6	5895	1.06 (1.01–1.11)	160,452.0	3857	1.08 (1.02–1.15)	144,472.5	1868	1.01 (0.96–1.07)	22,336.3	746	1.06 (0.98–1.16)
Physical activity (Yes)	281,482.8	4202	0.94 (0.90–0.98)	126,455.7	3009	0.93 (0.88–0.98)	141,473.8	2649	0.99 (0.99–1.04)	30,666.0	1201	0.94 (0.88–1.01)
	Residential area						Residential area					
	Urban			Rural			Urban			Rural		
Smoking status												
Past-smoker	49,388.9	1009	1.08 (1.01–1.17)	58,362.8	1299	1.11 (1.04–1.19)	3894.1	61	0.94 (0.73–1.21)	4304.9	94	1.11 (0.91–1.37)
Current-smoker	183,101.0	3155	1.13 (1.07–1.20)	221,541.9	3912	1.03 (0.98–1.08)	7846.4	180	1.06 (0.91–1.24)	7092.7	229	1.10 (0.96–1.25)
Drinking status (drinker)	248,831.1	4383	1.10 (1.04–1.16)	295,704.5	5369	1.05 (1.00–1.10)	85,201.4	1221	1.05 (0.98–1.12)	81,607.4	1393	1.01 (0.95–1.07)
Physical activity (Yes)	187,759.6	3429	0.97 (0.92–1.02)	220,178.9	3782	0.95 (0.91–0.99)	88,976.4	1929	0.97 (0.92–1.03)	83,976.4	1921	0.95 (0.93–1.04)
	Insurance						Insurance					
	Employee-insured			Self-insured			Employee-insured			Self-insured		
Smoking status												
Past-smoker	92,177.8	1728	1.10 (1.04–1.16)	15,573.9	580	1.09 (0.99–1.20)	6296.7	84	1.01 (0.81–1.26)	1902.3	71	1.07 (0.84–1.35)
Current-smoker	355,616.0	5359	1.07 (1.02–1.12)	49,026.9	1708	1.14 (1.06–1.23)	9306.2	170	1.11 (0.95–1.29)	5632.9	239	1.07 (0.94–1.22)
Drinking status (drinker)	478,844.1	7507	1.08 (1.03–1.12)	65,691.5	2245	1.04 (0.97–1.11)	138,422.5	1648	1.02 (0.97–1.09)	28,386.3	966	1.05 (0.98–1.13)
Physical activity (Yes)	365,715.6	5827	0.96 (0.93–1.00)	42,222.9	1384	0.91 (0.85–0.97)	128,483.0	2311	0.99 (0.94–1.04)	43,656.8	1539	0.95 (0.89–1.01)

HR, hazard ratio; CI, confidence interval; BMI, body mass index. Base groups: Smoking status (never-smoker), Drinking status (never-drinker), Physical activity (no). All models were adjusted for age, residential area, income level, insurance, BMI, family history of hypertension, other disease (diabetes, kidney, cancer), and drinking amount.

## Data Availability

All data and materials used in this study will be available upon reasonable request from the corresponding author.
